# Supplementary material for: MicroRNAs tend to synergistically control expression of genes encoding extensively-expressed proteins in humans
Source: PeerJ. 2017 Aug 14;5:e3682. doi: 10.7717/peerj.3682 (PMC5560240; doi:10.7717/peerj.3682)
Supplement: Table S1 [file peerj-05-3682-s001.docx]

**Table S1.** Sequences of the negative control and miRNA mimics used in this study.

| mimics | Sequence (5'-3') |
| --- | --- |
| negative control | UUUGUACUACACAAAAGUACUG |
| miR-1 | UGGAAUGUAAAGAAGUAUGUAU |
| miR-21 | UAGCUUAUCAGACUGAUGUUGA |
| miR-106a | AAAAGUGCUUACAGUGCAGGUAG |
| miR-133a | UUGGUCCCCUUCAACCAGCUGU |
| miR-142-5p | CAUAAAGUAGAAAGCACUACU |
| miR-155 | UUAAUGCUAAUCGUGAUAGGGGU |
