# Supplementary material for: MicroRNAs tend to synergistically control expression of genes encoding extensively-expressed proteins in humans
Source: PeerJ. 2017 Aug 14;5:e3682. doi: 10.7717/peerj.3682 (PMC5560240; doi:10.7717/peerj.3682)
Supplement: File S1 [file peerj-05-3682-s004.docx]

**A four-step protocol to reveal miRNAs that tend to regulate UEP-encoding mRNAs**

Step 1. Reliable miRNA-target interactions in humans were retrieved from three MTI databases including miRecords, miRSel, and ExprTargetDB.

Step 2. For each miRNA target gene, expression data of its protein product in 12 organs was retrieved from the human protein atlas (HPA) database. The 12 organs were breast and female reproductive system, blood and immune system, central nervous system, cardiovascular system, digestive tract, endocrine glands, liver and pancreas, male reproductive system, placenta, respiratory system, skin and soft tissues and urinary tract. NOTE: miRNAs that target less than 50 mRNAs in any one organ were excluded from this study.

Step 3. For each protein encoded by miRNA target gene, we converted the validated the HPA marks ‘none’, ‘low’, ‘medium’ and ‘high’ representing cellular expression levels into the digitals 0, 1, 2 and 3, respectively. Afterwards, coefficient of variation (CV) was calculated by using the Graphpad Prism software to evaluate the cellular expression dispersion degree of the given protein. NOTE: In this study, uniformly expressed proteins (UEPs) and disorderly expressed proteins (DEPs) were defined as proteins with CVs of less than 40% and CVs of more than 120%, respectively.

Step 4. For a miRNA, the statistics parameter skewness was calculated by using the Graphpad Prism software to evaluate the distribution inclination of all the log_2_CV values of proteins encoded by its target genes. Such an evaluation was organ-restricted. NOTE: A smaller skewness value (skewness < 0.3) means an obvious tendency of regulating UEP-encoding mRNAs.
